# The first description of a microtrombidiid mite (Actinotrichida: Prostigmata, Microtrombidiidae) from Baltic amber, with notes on related extant genera and species

**DOI:** 10.1007/s12542-016-0311-y

**Published:** 2016-05-30

**Authors:** Marta Konikiewicz, Elżbieta Sontag, Joanna Mąkol

**Affiliations:** 1 Department of Invertebrate Systematics and Ecology, Institute of Biology, Wroclaw University of Environmental and Life Sciences, Kożuchowska 5b, 51-631 Wrocław, Poland; 2 Museum of Amber Inclusions, Department of Invertebrate Zoology and Parasitology, University of Gdańsk, Wita Stwosza 59, 80-308 Gdańsk, Poland

**Keywords:** Succinite, Baltic amber, “Blue Earth”, Eocene, Acari, Parasitengona, Gulf of Gdańsk, Poland, Succinit, Baltischer Bernstein, “Blaue Erde”, Eozän, Acari, Parasitengona, Danziger Bucht, Polen

## Abstract

Discovery of parasitengone mites (Acari) in the Gulf of Gdańsk deposits of Baltic amber (“Blue Earth” sediment) resulted in the first description of a fossil representative of Microtrombidiidae. The new species, based on larvae, displays affinity to recent members of *Montenegtrombium* Saboori and Pešić, 2006, *Persianthrombium* Sedghi, Saboori and Hakimitabar (in Sedghi et al. 2010) and *Porttrombidium* Haitlinger, 2000, known from the southwestern Palaearctic. A comparison with related genera and species places the newly described taxon in *Porttrombidium* (as *Porttrombidium gedanense* sp. nov.). *Montenegtrombium* is regarded as a junior synonym of *Porttrombidium*.

## Kurzfassung

Die Entdeckung von Milben aus der Gruppe der Parasitengona (Acari) in Baltischem Bernstein aus der “Blauen Erde” der Danziger Bucht führt zur ersten Beschreibung eines fossilen Vertreters der Microtrombidiidae. Die Milbe ist als Larve konserviert und ähnelt rezenten Arten der Gattungen *Montenegtrombium* Saboori & Pešić, 2006, *Persianthrombium* Sedghi, Saboori & Hakimitabar (in Sedghi et al. 2010) und *Porttrombidium* Haitlinger, 2000, alle bekannt aus der südwestlichen Paläarktis. Ein Vergleich mit Arten dieser Gattungen zeigt, dass die neu nachgewiesene Bernstein-Larve als neue Art von *Porttrombidium* zu betrachten ist – *Porttrombidium gedanense* sp. nov. Bei der Gattung *Montenegtrombium* handelt es sich um ein jüngeres Synonym von *Porttrombidium*.

## Introduction

Baltic amber, also known as succinite, is widely distributed in central-eastern Europe. The richest and the oldest deposits are located within the Gulf of Gdańsk, at the mouth of the hypothetical Eridanus river, which brought the resin from the primary amber forest (Weitschat and Wichard 2002).

Research on amber inclusions has a history of more than 200 years (Perkovsky et al. 2007; Szwedo and Sontag 2009). Recent studies on the taxonomic grouping of zooinclusions in randomly selected pieces of Baltic amber revealed that mites, constituting more than 20 % of all zooinclusions, are one of the best represented groups in succinite, giving precedence only to Diptera, which account for ca. 40 % of inclusions (Sontag 2003). Despite the abundance of mites in Baltic amber, the knowledge of Eocene acarofauna is scant.

The cohort Parasitengona, comprising ca. 11,000 of the species described to date (ca. 5,000 terrestrial and ca. 6,000 aquatic), constitutes one of the most speciose groups of mites, with relatively scarce knowledge of fossil taxa. The first mention of terrestrial parasitengone amber inclusion originates from 1845 (Berendt 1845; Dunlop et al. 2015; Judson 2012), and relatively few species have been described till now (Dunlop et al. 2015; Konikiewicz and Mąkol 2014; Bartel et al. 2015). A summary of hitherto knowledge of fossil terrestrial parasitengones has been recently provided by Bartel et al (2015). The present work describes *Porttrombidium* *gedanense* sp. nov., based on larvae. It is the first representative of Microtrombidiidae found in the fossil record.

## Materials and methods

The samples belong to the Museum of Amber Inclusions (MAI), University of Gdańsk, Poland. Representatives of terrestrial Parasitengona mites were found in lumps of Baltic amber (reg. no. MAI 896, MAI 1343, MAI 3048) originating from the Gulf of Gdańsk deposits (incl. MAI 896 and MAI 1343: Sambia Peninsula, Kaliningrad Oblast, Russia). The lumps contained syninclusions (MAI 896: Homoptera; MAI 1343: Diptera: Dolichopodidae and Cecidomyiidae; MAI 3048: Staphylinidae: Pselaphinae (det. Daniel Kubisz), Coleoptera, Arachnida: Araneae, Myriapoda: Chilopoda).

In sample preparation we followed the protocol provided by Sidorchuk (2013). The amber pieces containing inclusions were pre-cut to the following dimensions (mm): 3.3 × 2.6 × 0.1 (MAI 896), 3.0 × 2.0 × 0.1 (MAI 1343) and 2.9 × 2.6 × 0.1 (MAI 3048), using a Dremel 300 rotary tool with a flexible drive and diamond disc. After polishing with a portable USB-powered MiniPolly2 polishing machine, the pieces were placed in cavity slides in thymol and distilled water solution and sealed with a cover glass. The inclusions were examined using a Nikon Eclipse E-600 light microscope, equipped with a DIC, drawing tube, and DS-Fi1 camera, at magnifications of 400× and 1000×. Raw drawings were graphically processed with the GIMP software, whereas the in-focus images were produced using CombineZP software. Measurements are given in micrometers. The samples are stored in Eppendorf vials filled with a solution of thymol and distilled water. The terminology follows Wohltmann et al. (2007) and Mąkol et al. (2014). For the purpose of comparison the type material of the following recent species was studied: *Porttrombidium sebastiani* Haitlinger, 2000 (holotype) and *Montenegtrombium baloutchi* Masoumi, Saboori and Seiedy, 2016 (four paratypes: MP 1256) deposited at the Museum of Natural History, University of Wrocław, Poland.

## Systematic palaeontology

Class Arachnida Cuvier, 1812

Superorder Actinotrichida Grandjean in van der Hammen, 1961

Order Trombidiformes Reuter, 1909

Suborder Prostigmata Kramer, 1877

Cohort Parasitengona Oudemans, 1909

Family Microtrombidiidae Thor, 1935

Genus *Porttrombidium* Haitlinger, 2000

*Montenegtrombium* Saboori and Pešić, 2006, syn. nov.

*Type species* *Porttrombidium sebastiani* Haitlinger, 2000, Recent, from *Calliptamus italicus* (L.) (Orthoptera) collected in Aire de Maire nr Fatima, Portugal.

*Diagnosis* larva. Microtrombidiinae with three unpaired idiosomal sclerites (scutum, scutellum, and postscutellum). Stolascutum absent. One pair of normal setae (*c*_1_) on scutellum and on postscutellum (*d*_1_). Stephanostome present. Hypostomalae simple. Palp femur with one seta, palp genu with 0–1 setae. Tarsi I–III with two claws and claw-like empodium, inner claw on tarsus III reduced to ca. 1/4 length of the outer claw. Additionally, elongated sword-like seta, similar in length to outer claw, present at tarsus III termination. Scopa and lophotrix absent. fCx = 2–2–1. Coxalae simple.

Deutonymph and adult. Not known.

*Remarks. * During the most recent re-examination of the type specimen of the type species (*Porttrombidium sebastiani* Haitlinger, 2000) we could observe that simple, setulated hypostomalae are present in the holotype, thus the character should be considered typical for the genus. The scope of differences observed between *Porttrombidium* and *Montenegtrombium*, pertaining to the chaetotaxy of the palps and chaetotaxy of tarsi, reflects the intra-generic variation known for other genera, and does not confirm their separate status. Consequently, we regard *Montenegtrombium* as a synonym of *Porttrombidium*. Masoumi et al. (2016) provided the verified characteristics of *Montenegtrombium* *milicae* Saboori and Pešić, 2006 (here regarded as *Porttrombidium milicae*) and pointed to the presence of ‘sword-like lophotrix’ in newly described *Montenegtrombium baloutchi* (here regarded as *Porttrombidium baloutchi*), whereas a sword-like seta arising at the tarsus III termination, and not being a lophotrix, is observed in the latter species but also in *P. gedanense* sp. nov. The lophotrix is absent in members of *Porttrombidium*.

*Porttrombidium gedanense* sp. nov.

Figures 1, 2, 3, 4, 5, 6, 7 and 8

**Fig. 1:**
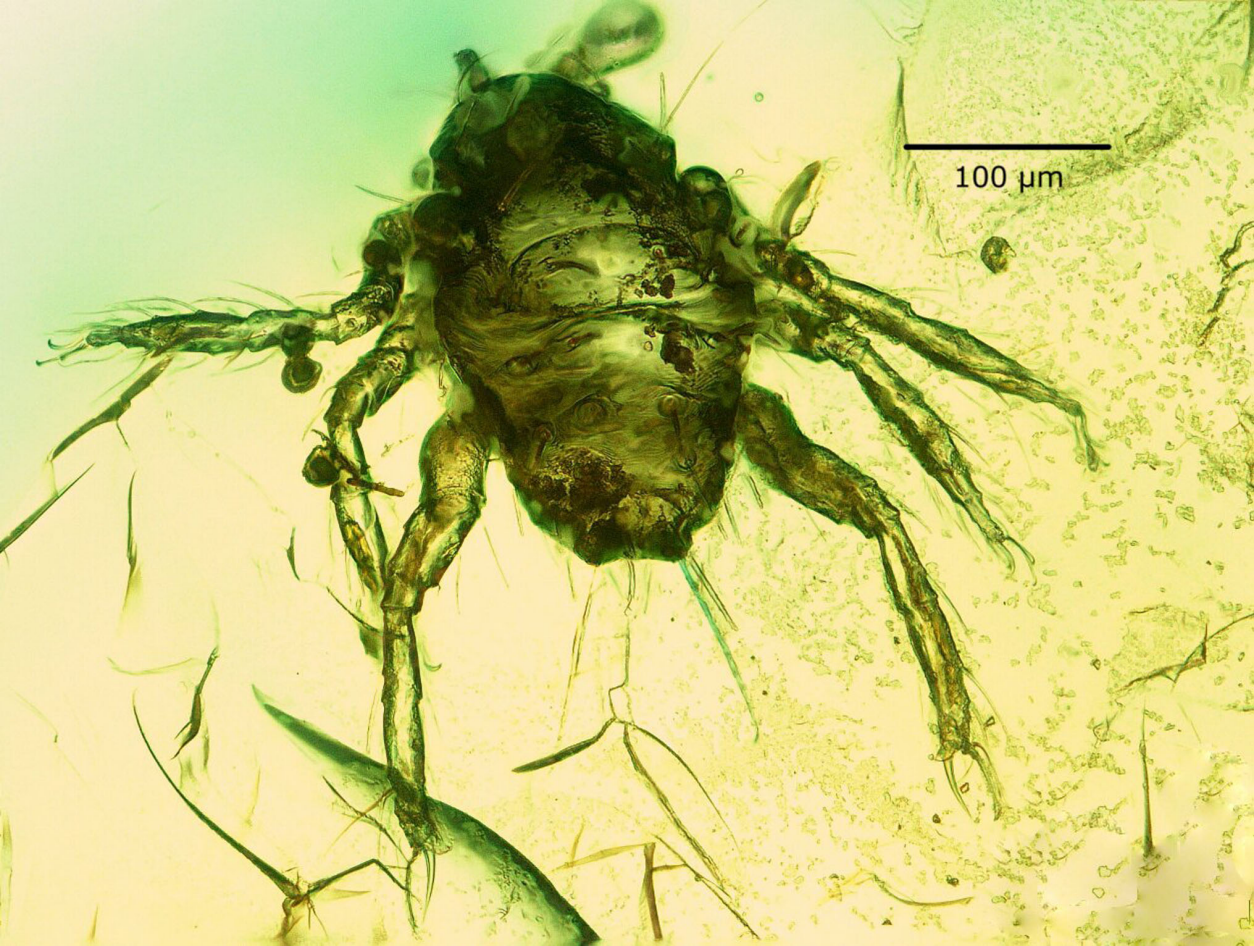
*Porttrombidium gedanense* sp. nov., larva (holotype, no. MAI 1343)

*Etymology.* The specific epithet refers to Gdańsk (Latin Gedanum), the Pomeranian city on the Gulf of Gdańsk, hosting the Museum of Amber Inclusions (MAI) (see also Type material) and located ca. 100 km away from amber deposits in the Sambia Peninsula.

*Type material. * Holotype (no. MAI 1343) and paratypes (no. MAI 896, no. MAI 3048) are deposited at the MAI, Department of Invertebrate Zoology and Parasitology, University of Gdańsk, Poland.

*Locality and horizon.* Holotype and paratypes originate from the Gulf of Gdańsk sediments (vic. of Jantarny, Sambia Peninsula, Kaliningrad Oblast, Russia), dated at Eocene (Lutetian), ca. 44–50 Ma (Bartel et al. 2015; Weitschat and Wichard 2002).

*Diagnosis* fnTi = 5–4–4, fnTa = 15–13–10. For other characters, see generic diagnosis.

*Description* holotype (reg. no. 1343), larva. Habitus as in Fig. 1. Metric data in Table 1.

**Table 1:** Metric data for the holotype and paratypes of *Porttrombidium gedanense* sp. nov. (measurements indicated in bold apply to structures positioned parallel to the amber piece surface)

	Holotype (no. MAI 1343)	Paratype (no. MAI 896)	Paratype (no. MAI 3048)
IL	243	**295**	**159**
IW	159	**148**	**119**
AW	67	**92**	**80**
PW	82	**85**	**82**
AA	107	**113**	**100**
SB	74	**74**	**83**
ASB	41	**122**	**118**
PSB	35	**30**	**47**
AP	45	**47**	**48**
MA	27	**60**	**55**
AL	28	–	–
PL	41	–	–
AM	56	–	–
S	68	–	–
SL	42	–	38
SS	54	**53**	53
LSS	96	**92**	85
HS	43	**36**	32
*h*_1_	**63**	53	39
*h*_2_	**101**	88	65
*bs*	17	–	–
1a	**23**	–	–
1b	**24**	–	–
2a	**20**	–	–
2b	**21**	–	–
3a	**21**	–	–
Cx I	**76**	66	57
Tr I	25	35	**47**
Fe I	38	33	**47**
Ge I	22	16	**25**
Ti I	**42**	36	**48**
Ta I (L)	**74**	50	**99**
Ta I (H)	**20**	30	**24**
Leg I	277	236	323
Cx II	**64**	59	**63**
Tr II	29.77	37.53	**52**
Fe II	27.57	43	**56**
Ge II	22	17	**25**
Ti II	**41**	26	33
Ta II (L)	**74**	36	60
Ta II (H)	**16**	24	26
Leg II	258	219	289
Cx III	**59**	55	**72**
Tr III	41	43	**73**
Fe III	45	25	**52**
Ge III	18	14	**30**
Ti III	**44**	25	**45**
Ta III (L)	**73**	42	**75**
Ta III (H)	**18**	29	**21**
Leg III	245	205	348
IP	780	659	959

Terminology after Wohltmann et al. (2007) and Mąkol et al. (2014)

**Figs. 2–4:**
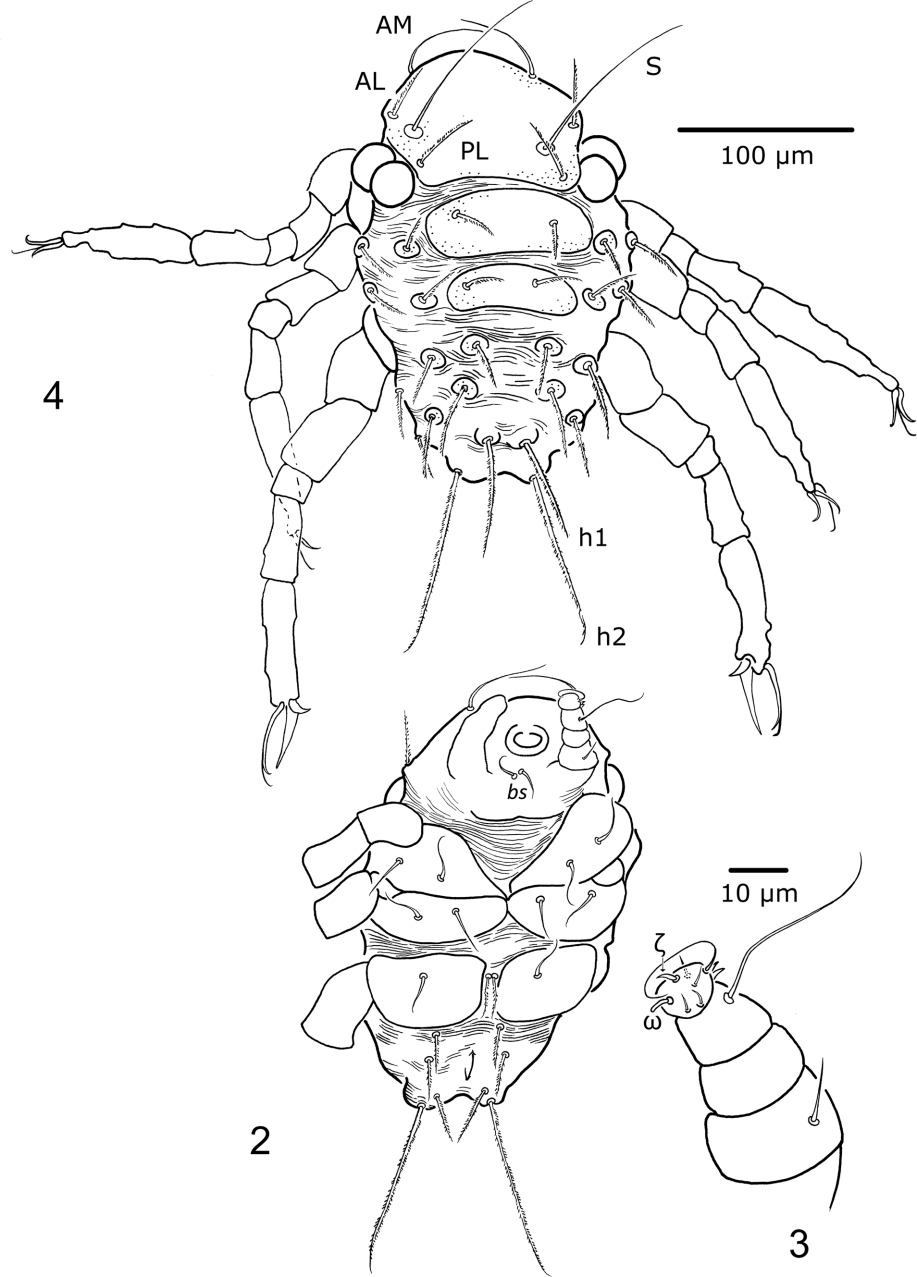
*Porttrombidium gedanense* sp. nov., larva (holotype, no. MAI 1343). **Fig. 2** Ventral aspect; **Fig.** **3** palp (femur–tarsus); **Fig.** **4** dorsal aspect

Gnathosoma (Figs. 2, 3). Stephanostome with horseshoe-like sclerite, devoid of lateral teeth. Hypostomalae (*bs*) slender, smooth (ca. 17). Palp trochanter and palp genu without setae. Palp femur with one smooth seta (12). On palp tibia only one elongated and smooth seta (37) is visible (see also Remarks). Odontus bifid at termination. Palp tarsus with four short and smooth setae, one slender, elongated and smooth seta and two sensillary setae (?*ω* and *ζ*) fPp = 0–*N*–0–[?][?]*N*–*NNNNN*?*ωζ*.

Idiosoma (Figs. 2, 4). Scutum, scutellum, and postscutellum porous. Scutum longer than wide, slightly incised at the level of ocular plates, with straight posterior margin, with three pairs of non-sensillary setae (AM, AL, PL) and one pair of smooth trichobothria (S). Setae AM arcuately bent, almost smooth. Scutellum with a pair of *c*_1_ setae, postscutellum with *d*_1_ setae. Setae AL, PL, *c*_1_ and *d*_1_ with distinct setules. Remaining setae on idiosoma dorsum, similar in shape to AL, PL, *c*_1_ and *d*_1_, placed on platelets, with stems gradually longer toward the idiosoma termination. Setae *h*_2_ distinctly elongated (101), less than twice as long as *h*_1_ (63). fD = (*c*_1_)*c*_2–3_–(*d*_1_)*d*_2–3_–*e*_1–3_–*f*_1–3_–*h*_1–2_. Ventrally on idiosoma six pseudanal setae, more slender than those covering the idiosoma dorsum. Oval Claparède’s organs between coxae I and II. Setae 1a and 1b—on coxa I, 2a and 2b—on coxa II and 3b—on coxa III. Setae 3a located between coxae III. All coxal and intercoxal setae similar in length, slender and [?] smooth.

**Figs. 5–8:**
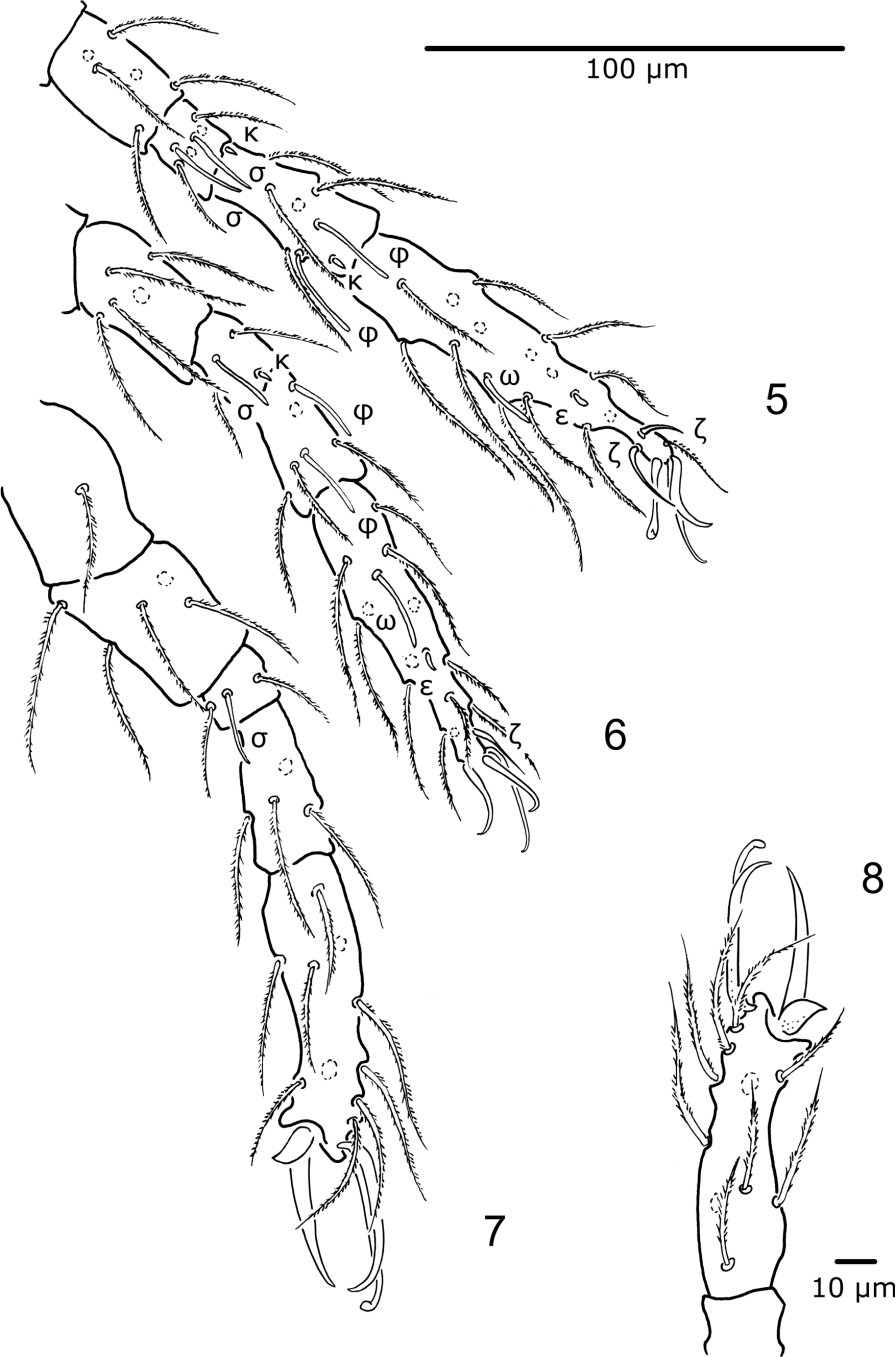
*Porttrombidium gedanense* sp. nov., larva (holotype, no. MAI 1343). **Fig.** **5** Leg I (femur–tarsus); **Fig.** **6** leg II (femur–tarsus); **Fig.** **7** leg III (trochanter–tarsus); **Fig.** **8** details of tarsus III

Legs (Figs. 5, 6, 7, 8) 6-segmented. Chaetotaxy: leg I—Cx 2*n* + *elc* I, Tr 1*n*, Fe 6*n*, Ge 4*n* + 2*σ* + 1*κ*, Ti 5*n* + 2*φ* + 1*κ*, Ta 15*n* + 2*ζ* + 1*ω* + 1*ε*; leg II—Cx 2*n*, Tr 1*n*, Fe 5*n*, Ge 2*n* + 1*σ* + 1*κ*, Ti 4*n* + 2*φ*, Ta 13*n* + 1*ζ* + 1*ω* + 1*ε*; leg III—Cx 1*n*, Tr 1*n*, Fe 5*n*, Ge 2*n* + 1*σ*, Ti 4*n*, Ta 10*n*. Tarsi I and II terminated with paired claws and claw-like empodium. Tarsus III with modified, markedly reduced inner claw, normally developed outer claw and empodium. Additionally, an elongated sword-like seta present at the Ta III termination.

*Remarks* the position of the specimen does not allow thorough examination of the chelicerae and setae *or* within the gnathosoma (the presence of *or* is typical of parasitengone larvae). Of three setae usually observed on the palp tibia, only one could be detected, due to the blurred surface of the segment. In the area surrounding the anus only six pseudanal setae could be observed (the actual number can depart from this value, however, the multiplication of setae can be excluded).

*Comparison* the new species differs from *Porttrombidium sebastiani* Haitlinger, 2000 in the chaetotaxy of tibia and tarsus I–III (fnTi = 5–4–4, fnTa = 15–13–10 in *P. gedanense*, fnTi = 6–6–6, fnTa = 22–16–17 in *P. sebastiani*) and in the number of dorsal setae [24 (+4) in *P. gedanense*, 22 (+4)—in *P. sebastiani*].

## Discussion

*Porttrombidium* Haitlinger, 2000, originally placed in Trombidiidae, was excluded from the nominate family by Mąkol (2007). Sedghi et al. (2010) placed the genus in Microtrombidiidae, and Mąkol and Wohltmann (2012) treated it as taxon *incertae sedis* within Microtrombidiinae. Six of the microtrombidiid genera (*Cercothrombium* Methlagl, 1928, *Keramotrombium* Southcott, 1994, *Persianthrombium* Sedghi, Saboori and Hakimitabar, 2010, *Porttrombidium* Haitlinger, 2000 (=*Montenegtrombium* Saboori and Pešić, 2006 syn. nov.), *Shibadania* Southcott, 1994, *Workandella* Southcott, 1994) have the third, postscutal shield, located medially on the idiosoma dorsum and encompassing the bases of the *d*_1_ setae (Haitlinger 2000; Methlagl 1928; Saboori and Pešić 2006; Sedghi et al. 2010; Southcott 1994). Another genus, *Crinitrombium* Southcott, 1994, having *d*_1_ plates fused, was synonymised with *Microtrombidium* by Gabryś and Wohltmann (2001). Of the above mentioned taxa, only *Keramotrombium*, *Persianthrombium*, and *Porttrombidium* (=*Montenegtrombium* syn. nov.), share the presence of two setae on coxa II and the distinctly reduced inner claw on the tarsus III termination. However, the same combination of characters is observed also in Achaemenothrombiidae Saboori, Wohltmann and Hakimitabar, 2010 and in some representatives of Trombidiidae Leach, 1815, thus the usefulness of these characters for phylogenetic inferences may be limited due to their homoplastic nature.

*Keramotrombium* Southcott, 1994 was erected in order to accommodate *Metathrombium argentanense* Bruyant, 1912. Another species, *Keramotrombium talebii* Karimi Iravanlou and Kamali, 2001, was originally placed in the genus by Karimi Iravanlou and Kamali (2001); however, after the re-appraisal of its characters, the species was transferred to *Achaemenothrombium* Saboori, Wohltmann and Hakimitabar, 2010. *Keramotrombium* differs from *Porttrombidium* also in having the distinct lateral teeth (the latter absent in *Porttrombidium*) within the stephanostome and in the presence of multiple pseudanal setae (a state not observed in *Porttrombidium*). Differences between *Porttrombidium* and *Persianthrombium* are expressed in the number of solenidia on the genu and tibia I (f_sol_I = 0–2–2–1 in *Porttrombidium*, f_sol_I = 0–4–4–1 in *Persianthrombium*).

Saboori et al. (2010) erected *Achaemenothrombium* Saboori, Wohltmann and Hakimitabar, 2010 and a new trombidioid family Achaemenothrombiidae Saboori, Wohltmann and Hakimitabar, 2010, to accommodate two species (among them *Keramotrombium talebii* Karimi Iravanlou and Kamali, 2001, described from Iran) with a combination of characters not known for any other family level taxon assigned to Trombidioidea: i.e. three dorsal scuta, fCx = 2–2–1, Ti I–III with eight or more normal setae, tibia I with at least four solenidia, multiple solenidia (at least four) and eupathidia (at least six) on tarsus I and multiple solenidia on tarsus II (at least two). The third species assigned to the family was described by Saboori et al (2013). Of the characters of Achaemenothrombiidae, the presence of three dorsal scuta, fCx = 2–2–1, but also the Ta III termination are shared by *Keramotrombium*, *Persianthrombium* and *Porttrombidium* (=*Montenegtrombium* syn. nov.).

Both *P. sebastiani* and *P. milicae* were recorded from *Calliptamus italicus* (L.) (Orthoptera: Acrididae), *P. milicae* was recorded also from *Carpocoris purpureipennis* (De Geer) (Hemiptera: Pentatomidae), whereas *P. baloutchi*—from *Acrida* sp., *Oedipoda schochii* Brunner von Wattenwyl and *Chorthippus brunneus* (Thunberg) (Orthoptera: Acrididae) (Haitlinger 2000; Masoumi et al. 2016; Saboori and Pešić 2006). *Persianthrombium* has been hitherto known to parasitize *Locusta* sp. (Orthoptera: Acrididae) (Sedghi et al. 2010), whereas the host of *Keramotrombium* remains unknown (Bruyant 1912). Larvae of Achaemenothrombiidae parasitize Lepidoptera and Orthoptera (Saboori et al. 2010, 2013).

The recent species of the above-mentioned genera, tentatively assigned to Microtrombidiidae and of Achaemenothrombiidae have been recorded from Portugal (*Porttrombidium*), France (*Keramotrombium*), Montenegro (*Montenegtrombium* = *Porttrombidium*), and Iran (Achaemenothrombiidae and *Persianthrombium*). The discovery of the new fossil member of *Porttrombidium* from the Gulf of Gdańsk amber deposits may support the hypothesis of similar ecological preferences shared by the recent taxa and inhabitants of the amber forest.

Microtrombidiinae may include genera of heterogeneous origin. A discussion on constructing monophyletic groups (subfamilies) within Microtrombidiidae, supported by a critical review of the literature data, was provided by Wohltmann (2006). The ultimate answer to the question of monophyly of these groups constitutes a crucial point in further conclusions on the phylogeny of subordinate taxa and their position in the system of Microtrombidiidae.

*Porttrombidium* shares the characters known for Microtrombidiidae (e.g. stephanostome) and for Trombidiidae (e.g. fCx = 2–2–1, termination of tarsus III). The systematic position of *Porttrombidium* but also of *Persianthrombium*, and their relationship with other microtrombidiid genera and with Achaemenothrombiidae and Trombidiidae, should be clarified based on further evidence from biology and genetic studies. Discovery of postlarval forms, hitherto unknown both for *Porttrombidium*, *Keramotrombium*, *Persianthrombium* and for Achaemenothrombiidae, may shed a new light on the picture of relationships within Trombidioidea.
